# The Inhibition of Prolyl Oligopeptidase as New Target to Counteract Chronic Venous Insufficiency: Findings in a Mouse Model

**DOI:** 10.3390/biomedicines8120604

**Published:** 2020-12-13

**Authors:** Giovanna Casili, Marika Lanza, Sarah Adriana Scuderi, Salvatore Messina, Irene Paterniti, Michela Campolo, Emanuela Esposito

**Affiliations:** Department of Chemical, Biological, Pharmaceutical and Environmental Sciences, University of Messina, 98166 Messina, Italy; gcasili@unime.it (G.C.); mlanza@unime.it (M.L.); sarascud@outlook.it (S.A.S.); smessina23@gmail.com (S.M.); ipaterniti@unime.it (I.P.); campolom@unime.it (M.C.)

**Keywords:** chronic venous insufficiency, prolyl oligopeptidase (POP), inflammation, angiogenesis, endothelial disfunction

## Abstract

(1) Background: Chronic venous insufficiency (CVI) is a common disorder related to functional and morphological abnormalities of the venous system. Inflammatory processes and angiogenesis alterations greatly concur to the onset of varicose vein. KYP-2047 is a selective inhibitor of prolyl oligopeptidase (POP), a serine protease involved in the release of pro-angiogenic molecules. The aim of the present study is to evaluate the capacity of KYP-2047 to influence the angiogenic and inflammatory mechanisms involved in the pathophysiology of CVI. (2) Methods: An in vivo model of CVI-induced by saphene vein ligation (SVL) and a tissue block culture study were performed. Mice were subjected to SVL followed by KYP-2047 treatment (intraperitoneal, 10 mg/kg) for 7 days. Histological analysis, Masson’s trichrome, Van Gieson staining, and mast cells evaluation were performed. Release of cytokines, nitric oxide synthase production, TGF-beta, VEGF, α-smooth muscle actin, PREP, Endoglin, and IL-8 quantification were investigated. (3) Results: KYP-2047 treatment ameliorated the histological abnormalities of the venous wall, reduced the collagen increase and modulated elastin content, lowered cytokines levels and prevented mast degranulation. Moreover, a decreased expression of TGF-beta, eNOS, VEGF, α-smooth muscle actin, IL-8, and PREP was observed in in vivo study; also a reduction in VEGF and Endoglin expression was confirmed in tissue block culture study. (4) Conclusions: For the first time, this research, highlighting the importance of POP as new target for vascular disorders, revealed the therapeutic potential of KYP-2047 as a helpful treatment for the management of CVI.

## 1. Introduction

Chronic venous insufficiency (CVI) is a debilitating condition whose manifestations are extremely prevalent and reported to affect up to 80% of the population [1], with a varying percentage of incidences from 2 to 56% in men and 1 to 60% in women [2,3]; advanced age, obesity, and a positive family history represent the most important risk factors for developing this chronic venous disease [4]. The pathophysiology is complex, with a wide clinical spectrum, ranging from asymptomatic but cosmetic problems to severe symptoms; this includes telangiectases, reticular veins, varicose veins, edema, pigmentation and/or eczema, lipodermatosclerosis, and venous ulceration [5]. Genetic susceptibility and environmental factors affect CVI, generating a concerning symptomatology: pain, dermal irritation, swelling, skin changes, associated with a real risk of developing debilitating venous ulceration. The increase in venous pressure referred to as venous hypertension symbolizes the main signature of this venous pathology [2]. The symptomatology correlated to CVI results in various clinical signs that may severely compromise life quality, carrying out the importance of a speedy recognition to provide symptomatic relief and prevent pathology progression [6]. CVI is a relatively common health problem but is often ignored by healthcare providers because of an underappreciation of the magnitude and impact of the problem, as well as incomplete recognition of the various presenting manifestations of primary and secondary venous disorders [7]. There is a wide range of clinical options—both conservative and invasive—for the treatment of CVI, united by a single goal, which is to improve the symptomatology and to prevent sequelae and complications, promoting the ulcer healing [8,9,10]. Despite great therapeutic advances, there is to date no intervention that can definitively prevent or resolve the recurrence of this chronic venous disease. Hence there is a need to better understand the focal points of the pathogenetic process in order to find better therapeutic strategies.

Angiogenesis is crucial in the formation of collateral vessels as part of an adaptive response to vascular occlusion and ischemia, playing an important role in conditions including vascular diseases; conversely, excessive pathological angiogenesis driven by inflammation is a key contributor to the development and progression of cardiovascular-related disorders, representing an unfavorable process in venous system disorders [11,12]. It is known that angiogenesis inhibition could significantly impact the tissue breakdown and could hereby enable the formation of venous ulcerations [13]. In turn, activated leukocytes can release a large amount of elastase and other proteinases associated with tissue injury and lipodermatosclerotic skin remodeling. Specifically, the phenomenon of the “leucocyte trap” is of great interest, suggesting the fundamental importance of inflammation in the pathogenesis of venous insufficiency [14,15].

Prolyl oligopeptidase (POP), also known as propyl endopeptidase, is a serine protease involved in the release of pro-angiogenic and anti-fibrogenic molecules. Particularly, POP is present in all organs, localized in specific cells and cell layers across the brain and peripheral tissues (as skeletal muscle, testis, liver, kidney, lung, renal cortex, gut) and it is implicated in the hydrolysis of proline-containing bioactive peptides, such as angiotensins, arginine-vasopressin, substance P, and neurotensin [16,17]; it has also been shown to be involved in several other physiological and pathological functions such as inflammation [18].

Several potent substrate-like POP inhibitors have been developed and 4-phenyl-butanoyl-l-prolyl-2(S)-cyanopyrrolidine (KYP-2047) appears to be the most potent and widely studied in vitro and in vivo model. The aim of the present study was to evaluate the capacity of KYP-2047 to influence the angiogenic and inflammatory mechanisms involved in the pathophysiology of CVI, to restore normal vascular blood flow. Particularly, anti-inflammatory and anti-angiogenic activities of treatment with KYP-2047 were evaluated through an in vivo mouse model of CVI performed by saphene-vein ligation and confirmed in a tissue block culture study.

## 2. Materials and Methods

### 2.1. Animals

Male NMRI mice (12 weeks old) were obtained from Charles River and housed under specific pathogen-free conditions with the approval of the local animal ethics and welfare committee. The animals were maintained in a 12-h light–dark cycle and were provided with food and water at libitum. Animal experiments are in compliance with Italian regulations on protection of animals used for experimental and other scientific purposes (DM 116192) as well as EU regulations (OJ of EC L 358/1 12/18/1986) and ARRIVE guidelines. This study was approved by the University of Messina Review Board for the care of animals in compliance with Italian regulations on protection of animals (n 499/2018-PR released on 23 February 2018).

### 2.2. Saphene Vein Ligation (SVL) Model

All animals were treated under general anesthesia and oxygenated with a mechanical respirator. The animal was placed in the lateral supine position on the operating table. The hind legs were shaved, the skin was disinfected with iodine, and sterile draping was applied. A small transverse skin incision at the level of the ankle was performed, to exhibit underlying vasculature the lateral saphenous vein; it was chosen because of similarity to the human saphenous vein regarding diameter and length. The ligation was performed as previously described [19]. Proximally and distally the lateral saphenous vein was ligated with a Vicryl 3-0 suture (Johnson & Johnson, New Brunswick, NJ, USA). Long ends of the suture were used for identification of the proximal end. Large side branches were ligated with a similar suture or with a titanium clip. The veins were surgically exposed over the total length of treatment and monitored macroscopically for occlusion or any complication (perforation, rupture, or vein wall hematoma).

### 2.3. Experimental Groups

Mice were divided into six experimental groups:

Group 1: Sham + vehicle, control group to which the saphenous vein ligation was not performed, orally administered with saline for 7 days (n = 8);
Group 2: Sham + Simvastatin, group to which the saphenous vein ligation was not performed, orally administered with Simvastatin (20 mg/kg) for 7 days (n = 8);
Group 3: Sham + KYP-2047 group to which the saphenous vein ligation was not performed; KYP-2047 (10 mg/kg) was intraperitoneal (i.p.) administered for 7 days (n = 8);
Group 4: Saphene vein ligation (SVL), group subjected to ligation of the saphenous vein, orally administered with saline for 7 days (n = 8);
Group 5: SVL + Simvastatin, group subjected to ligation of the saphenous vein, orally administered with Simvastatin (20 mg/kg), 30 min after saphene vein ligation, for 7 days (n = 8);
Group 6: SVL + KYP-2047, subjected to ligation of the saphenous vein, i.p. administered with KYP-2047 (10 mg/kg), 30 min after saphene vein ligation, for 7 days (n = 8)

KYP-2047 was dissolved in saline containing 5% of Tween 80 and administered at the recommended dose of 10 mg/kg, according to the bibliography [20]. Moreover, mice were treated with Simvastatin because statins represent a potential pharmacological treatment option suitable to prevent growth of varicose veins and to limit the formation of recurrence after varicose vein surgery [21]. Based on animal weight and ingestion, the applied dose of Simvastatin was 20 mg/kg per day.

### 2.4. Histological Analysis

Seven days after the surgery, mice were sacrificed and lateral saphene veins were collected for histopathological examination and standard hematoxylin and eosin (H&E) staining was performed. Briefly, lateral saphene veins were before fixed in 10% (*w*/*v*) PBS-buffered formalin and then 7-µm sections were prepared from paraffin-fixed tissues. Following dehydration with ethanol and embedding with paraffin, 7 µm sections were made, followed by H&E staining. Based on the knowledge of the histopathology of varicose vein disorder [22], the following morphological criteria were considered to perform the histological score: score = 0, no structural or morphological damage to the three layers of the vessel wall; score = 1, slight morphological alteration; score = 2, dilation of the vessel lumen and structural alteration of the vessel wall; score = 3, hypertrophy of the tunica media, followed by slight neutrophilic accumulation; score = 4, structural alteration of the tunica adventitia and formation of focal epithelial edema with narrowing of the vessel lumen; score = 5, high presence of neutrophilic infiltrate and collapse of the whole vessel wall. The sections were evaluated by computer-assisted color image investigation (Leica QWin V3, Cambridge, UK). The histological results were showed 20× (50 μm of the Bar scale).

### 2.5. Masson Trichrome Staining

To evaluate the degree of fibrosis, tissue sections from saphene veins were stained with Masson trichrome according to the manufacturer’s protocol (Bio-Optica, Milan, Italy), as previously described [23]. Particularly, the entire area represented by the three tunicae, surrounding the veins, was considered for the quantification of the collagen in each section.

### 2.6. Van Gieson Staining

To detect the elastic fibers, tissue sections from saphene veins were stained with Elastica van Gieson staining kit, according to the manufacturer’s protocol (#115974, Sigma-Aldrich, St. Louis, MO, USA), as previous described [24].

### 2.7. Toluidine Blue Staining

For evaluation of the number and degranulation of mast cells, tissue sections from saphene veins were stained with toluidine blue (#05-M23001, Bio-Optica, Milan, Italy) as previously described [25].

### 2.8. Western Blot Analysis for Interleukin 1β (IL-1β), Tumor Necrosis Factor α (TNF-α), Transforming Growth Factor β (TGFβ1), Vascular Endothelial Growth Factor (VEGF), α-Smooth Muscle Actin (αSMA) and Prolyl Endopeptidase

Total cytosolic and nuclear extracts were prepared, as previously described [26], on saphene veins. The following primary antibodies were used: anti- IL-1β (Santa Cruz Biotechnology, Dallas, TX, USA, 1:500 #sc12742, D.B.A, Milan, Italy), anti-TNF-α (Santa Cruz Biotechnology; 1:500 #sc52746), anti-TGFβ1 (Santa Cruz Biotechnology, 1:500 #sc130348, D.B.A, Milan, Italy), anti-VEGF (Santa Cruz Biotechnology; 1:1000 #sc7269), anti- αSMA (Santa Cruz Biotechnology; 1:500 #sc53015) and anti-prolyl endopeptidase (Abcam; 1:1000,#ab58988) in 1× phosphate-buffer saline (Biogenerica srl, Catania, Italy), 5% *w/v* non-fat dried milk, 0.1% Tween-20 at 4 °C overnight. Membranes were incubated with peroxidase-conjugated bovine anti-mouse IgG secondary antibody (Jackson ImmunoResearch, West Grove, PA, USA; 1:2000) for 1 h at room temperature. Anti-β-actin (Santa Cruz Biotechnology; 1:1000 #sc47778) and anti-βTubulin (Santa Cruz Biotechnology; 1:1000 #sc5274) antibodies were used as controls. Protein expression was detected by chemiluminescence (ECL) system (Thermo, Waltham, MA, USA), visualized with the ChemiDoc XRS (Bio-Rad, USA), and analyzed by using Image Lab 3.0 software (Bio-Rad, Hercules, CA, USA) as previously reported [27].

### 2.9. ELISA Kit Assay for eNOS and IL-8, Pro-Collagen 1 Alpha, and TGFβ1

ELISA kit assay for endothelial nitric oxide synthase (eNOS) and interleukin 8 (IL-8) were performed respectively on saphene vein samples, as previously described [28]. In details, samples were thawed on ice and homogenized in 300 μL lysis buffer (750 μL, Pierce #87787, Thermo Fisher Scientific, Waltham, MA, USA) supplemented with a protease inhibitor cocktail (Sigma-Aldrich, Rehovot, Israel). Thereafter, the samples were homogenized and centrifuged at 14,000× *g* for 10 min at 4 °C; supernatants were collected, aliquoted, and stored at −20 °C. eNOS, IL-8, Pro-collagen 1 alpha, and TGFβ-1 were measured by ELISA kits according to the manufacturer’s instructions. The following kits, for mouse protein identifications, were used: mouse eNOS ELISA Kit (ab230938; Abcam, Cambridge, UK), mouse IL-8 ELISA Kit (MBS7606860; MyBiosource, San Diego, CA, USA), mouse Pro-Collagen I alpha 1 ELISA Kit, (ab229425; Abcam), and mouse TGF beta 1 ELISA Kit (ab119557; Abcam).

### 2.10. Myeloperoxidase (MPO) Activity

Veins tissues were analyzed for myeloperoxidase (MPO) activity, an indicator of polymorphonuclear leukocyte accumulation, using a spectrophotometric assay with tetramethylbenzidine as substrate, according to a method previously described [29]. MPO activity, as the quantity of enzyme degrading 1 μmol of peroxide per min at 37 °C, was expressed in U/g wet tissue.

### 2.11. Primary Culture of Vascular Smooth Muscle Cells (VSMCs) from Murine Saphene Vein: Tissue Block Culture Study

To demonstrate the compatibility of the in vivo model experiment, a study from tissue block culture was performed, as previously described [30]. The procedure for the experimental method includes the following steps: replication of in vivo study, isolation of the saphene vein, removal of the fat tissue around the vein, separation of the media, cutting the media into small tissue blocks, transferring the tissue blocks to cell culture plates, and incubation until the cells reach confluence.

#### 2.11.1. Experimental Groups for In Vivo Study

Mice were divided into three experimental groups:

Group 1: Sham + vehicle, control group to which the saphenous vein ligation was not performed, orally administered with saline for 7 days (n = 8);
Group 2: Saphene vein ligation (SVL), group subjected to ligation of the saphenous vein, orally administered with saline for 7 days (n = 8);
Group 3: SVL + KYP-2047, group subjected to ligation of the saphenous vein, administered with KYP-2047 (i.p., 10 mg/kg), 30 min after saphene vein ligation, for 7 days (n = 8).

After the sacrifice, saphene veins samples from each mouse, were picked up and longitudinally cut and placed in another cell culture dish containing DMEM. After removal of the fat tissue around the vein, the media tunica was extracted from the vein by pressing and pushing the vein with its blunt back side. Then, the media was cut into approximately 1-mm squares and transferred into cell culture. DMEM containing 20% FBS was carefully added and the tissue blocks were incubated in cell culture chamber for 5 days. The cells were identified as vascular smooth muscle cells (VSMCs). VSMCs obtained from vein samples of each experimental group were processed for immunofluorescence analysis and ELISA kit detection.

#### 2.11.2. Immunofluorescence Analysis

Immunofluorescence analysis was performed as previously described [31]. VSMCs were plated (1 × 10^4^/well) on glass coverslips in a culture dish of size 100 mm. After 24 h of adhesion at 37 °C and 5% CO_2_, cells were fixed in 4% paraformaldehyde and rinsed briefly in phosphate-buffered saline (PBS: 0.15 M NaCI, 10 mM Na_2_HPO_4_, 3 mM NaN_3_, pH 7.4), permeabilized in 0.2% Triton X-100/PBS and blocked with 10% goat serum. The cells were stained with an antibody against VEGF (1:200, Monoclonal Antibody JH121; Thermofisher, USA) O/N, followed by ITC-conjugated anti-mouse Alexa Fluor-488 antibody (1:2000 *v/v* Molecular Probes, UK) for 1 h at 37 °C. Sections were washed and for nuclear staining 4′,6′-diamidino-2-phenylindole (DAPI; Hoechst, Frankfurt; Germany) 2 μg/mL in PBS was added. Sections were observed and photographed at ×40 magnification using a Leica DM2000 microscope (Leica).

#### 2.11.3. ELISA Kit for Endoglin

ELISA kits assay for and Endoglin (CD105) was performed on primary cells VSMCs obtained from vein samples, as previous described [28]. In details, cell culture supernatants collected from VSMCs after 24 h of adhesion at 37 °C and 5% CO_2_, were thawed on ice, aliquoted, and stored at −20 °C. The following kit, for mouse protein identifications, was used: Endoglin/CD105 ELISA Kit (ab240677; Abcam, Cambridge, UK).

### 2.12. Materials

KYP-2047 (Sigma, cat#SML0208, Lot#032M4606V) was obtained by Sigma-Aldrich (Milan, Italy). Unless otherwise stated, all compounds were obtained from Sigma-Aldrich (St. Louis, MO, USA). All other chemicals were of the highest commercial grade available. All stock solutions were prepared in non-pyrogenic saline (0.9% NaCl, Baxter, Milan, Italy).

### 2.13. Statistical Analysis

All values are showed as mean ± standard error of the mean (SEM) of N observations. N denotes the number of animals employed. The experiment is representative of at least three experiments performed on different days on tissue sections collected from all animals in each group. Data were analyzed with GraphPad 5 software, by one-way ANOVA followed by a Bonferroni post-hoc test for multiple comparisons. A *p*-value of less than 0.05 was considered significant.

## 3. Results

### 3.1. The Expression of POP in CVI Mouse Model

**Figure 1:**
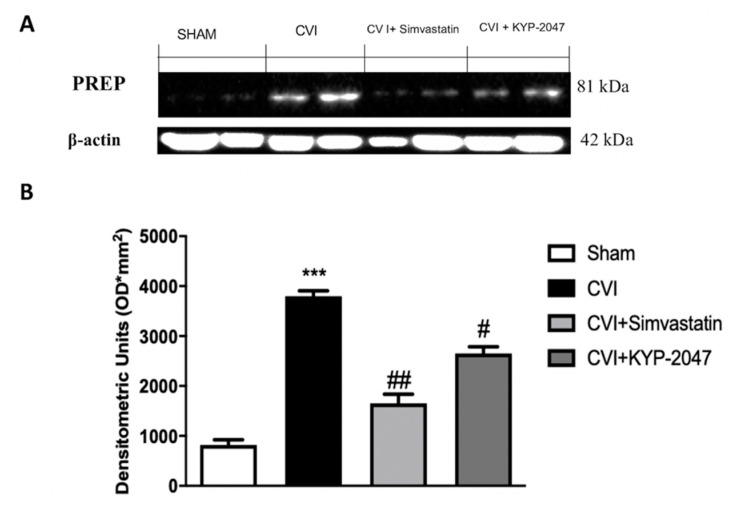
The inhibitor effects of KYP-2047 on prolyl oligopeptidase (POP) in chronic venous insufficiency (CVI) mouse model. Western Blot analysis to detect PREP in vein samples, clearly demonstrated the increase of POP in CVI insufficiency (**A**,**B**) compared to the control group (**A**,**B**). Moreover, this study confirmed the inhibitory role of KYP-2047 on POP enzymatic activity, also demonstrating an important inhibition effect on POP expression (**A**,**B**). Data represent the means of at least three independent experiments. One-way ANOVA followed by Bonferroni post-hoc. *** *p* < 0.001 versus Sham and ^##^ *p* < 0.01 and *^#^ p* < 0.05 versus CVI.

To clearly demonstrate the role of POP in CVI insufficiency, we performed a Western Blot analysis to detect PREP in vein samples. A basal expression on PREP was observed in samples from control group (Figure 1A,B) compared to the significant increase observed in samples from CVI-injured group (Figure 1A,B), for the first time demonstrating an over expression of PREP in these venous pathology. Moreover, small peptides structure-activity studies have shown that POP covalent inhibitors, as KYP-2047, are more potent and effective than their non-covalent analogs [32] because of the transient covalent bond with the enzyme that is hydrolyzed after a short time. In this study we confirmed the inhibitory role of KYP-2047 on POP enzymatic activity, also demonstrating an important inhibition effect on POP expression (Figure 1A,B).

### 3.2. Role of KYP-2047 Treatment on TGF-β1 and IL-8, as Vascular Markers in CVI

**Figure 2:**
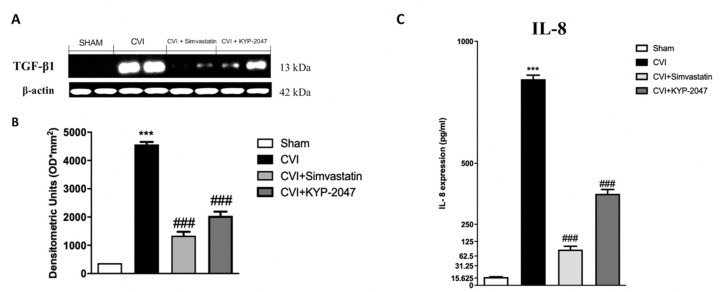
KYP-2047 treatment on TGF-β1 expression and IL-8 content. TGF-β1 expression was analyzed by Western Blot, suggesting an increment of this marker in CVI group, compared to control animals (**A**), see the densitometric units score (**B**); treatment with KYP-2047 significantly reduced TGF-β1 expression (**A**), see the densitometric units score (**B**). ELISA kit for IL-8 expression on saphene vein samples was performed; treatment with KYP-2047 (10 mg/kg, i.p.) significantly reduced IL-8 quantification (**C**), compared to the high amount of IL-8 released in the vein samples subjected to damage (**C**). Data represent the means of at least three independent experiments. One way ANOVA followed by Bonferroni post-hoc. *** *p* < 0.001 versus Sham; ^###^ *p* < 0.001 versus CVI.

An important mediator in CVI is represented by TGF-β1, a highly complex polypeptide involved in venous pathophysiology [33]; particularly, TGF-β1 contributes to specific pathological processes concerning the vessel wall [34], participating in vascular pathologies associated with matrix remodeling and fibrosis [35]. In this research, TGF-β1 expression levels were analyzed by Western Blot, suggesting an increment of this marker in SVL group, compared to control animals (Figure 2A, see the densitometric units score Figure 2B). Treatment with KYP-2047 significantly reduced TGF-β1 expression (Figure 2A, see the densitometric units score Figure 2B). GF-β1 is a pleiotropic factor that plays pivotal roles in angiogenesis and thus is indispensable for the development and homeostasis of the vascular system [36]. Moreover, varicose veins had a distinct chemokine expression pattern, since significant up-regulation of IL-8 [37], an angiogenic chemokine produced by a variety of cell types [38]. To understand the modulation of KYP-2047 treatment on IL-8 expression, we performed an ELISA kit for IL-8 on vein samples, observing a notable reduction on IL-8 expression in samples treated with KYP-2047 compared to CVI-damaged group (Figure 2C).

### 3.3. Role of KYP-2047 Treatment on Angiogenesis Modulation and Vasodilation

**Figure 3:**
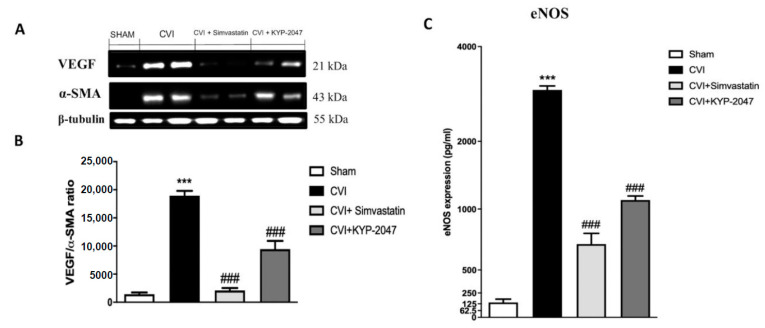
KYP-2047 treatment on VEGF/α-SMA expression and eNOS levels. VEGF/α-SMA ratio expression was analyzed by Western blot, suggesting an increment of this marker in CVI group, compared to control animals (**A**), see the densitometric units score (**B**); treatment with KYP-2047 significantly reduced VEGF/α-SMA expression (**A**), see the densitometric units score (**B**). ELISA kit for eNOS expression on saphene vein samples was performed; treatment with KYP-2047 (10 mg/kg, i.p.) significantly reduced IL-8 quantification (**C**), compared to the high amount of eNOS released in the CVI-damaged groups (**C**). Data represent the means of at least three independent experiments. One-way ANOVA followed by Bonferroni post-hoc. *** *p* < 0.001 versus Sham; ^###^ *p* < 0.001 versus CVI.

To highlight the in vivo modulatory action of KYP-2047 on angiogenesis, a Western blot analysis was performed to quantify VEGF and α-SMA expression on vein samples. In details, α-SMA, an isoform of the vascular smooth muscle actins, typically expressed in the vascular smooth muscle cells contributing to vascular motility and contraction, was found to be increased in varicose veins [39]; while VEGF plays an important role in maintaining the integrity of blood vessel walls [7]. VEGF/α-SMA ratio expression levels increased in CVI group, compared to control animals (Figure 3A, see the densitometric units score Figure 3A); treatment with KYP-2047 significantly reduced VEGF/α-SMA expression (Figure 3A, see the densitometric units score Figure 3B), like Simvastatin group (Figure 3A, see the densitometric units score Figure 3A). VEGF primarily exerts its effect through the production of vasodilatory mediators. VEGF signaling through VEGFR increases nitric oxide (NO) production, acutely bring to the eNOS activation [40]. The resultant increase in NO production promotes vascular permeability and endothelial cell survival, because NO also diffuses to adjacent vascular smooth muscle cells and mediates endothelium-dependent vasodilation [41]. To better understand the capacity of KYP-2047 treatment in vessel remodeling trough endothelium-derived nitric oxide, ELISA kit for eNOS expression on saphene vein samples was performed. Interestingly, treatment with KYP-2047 (10 mg/kg, i.p.) significantly reduced eNOS quantification (Figure 3C), compared to the high amount of eNOS released in the vein samples subjected to damage (Figure 3C).

### 3.4. Treatment with KYP-2047 on Cytokines Expression in SVL-Damaged Mice

**Figure 4:**
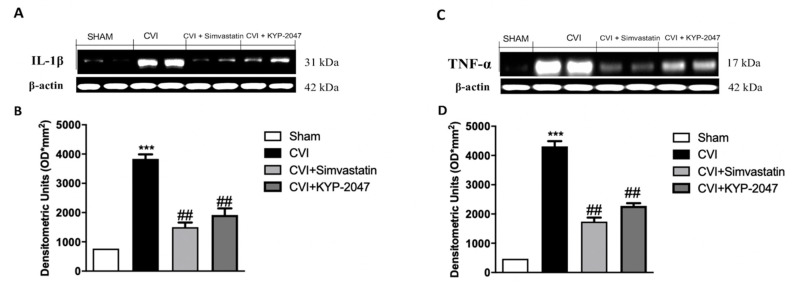
Treatment with KYP-2047 on cytokines expression. IL-1β and TNF-α expression levels, monitored by Western blotting in saphene veins tissue, were increased in CVI group, compared to sham basal levels respectively (**A**), see the densitometric units score (**B**,**C**), see the densitometric units score (**D**). KYP-2047 administration (10 mg/kg, i.p.) significantly reduced both cytokines proteins expression, similar to the election treatment represented by Simvastatin (20 mg/kg, oral) respectively (**A**), see the densitometric units score (**B**,**C**), see the densitometric units score (**D**). Data represent the means of at least three independent experiments. One-way ANOVA followed by Bonferroni post-hoc. *** *p* < 0.001 versus Sham; ^##^ *p* < 0.01 versus CVI.

Recent findings indicate that inflammatory processes are crucial for the development of incompetent valves and vein wall remodeling in CVI [42]. In this study, both IL-1β and TNF-α expression levels, assessed by Western blotting in saphene veins tissue, were increased in CVI group, compared to sham basal levels (respectively Figure 4A, see the densitometric units score Figure 4B,C, see the densitometric units score Figure 4D). KYP-2047 administration (10 mg/kg, i.p.) significantly reduced both cytokines proteins expression, similar to the election treatment represented by Simvastatin (20 mg/kg, oral) (respectively Figure 4A, see the densitometric units score Figure 4B,C, see the densitometric units score Figure 4D).

### 3.5. Effects of KYP-2047 on Histological Damage and Neutrophilic Activation Induced by SVL in Mice

**Figure 5:**
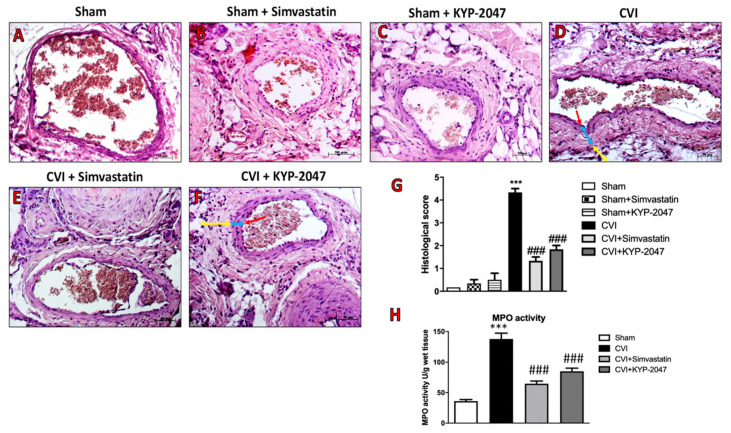
Treatment with KYP-2047 on histological damage and MPO activity induced by SVL in mice. Histopathologic examination, by hematoxylin-eosin staining, of lateral saphene vein subjected to ligation for 7 days (**D**,**G**) revealed an improvement in saphene vein treated with KYP-2047 (**F**,**G**) or Simvastatin (**E**,**G**). No damage or structural vein wall modification were observed in control groups (**A**–**C**,**G**). In particular, we detected tunica intima (red line), tunica media (blue line), tunica adventitia (yellow line), showing as treatment with KYP-2047 significantly reduced the hypertrophy of tunica media and preventing the collapse of the wall caused by the ligation (**F**,**G**). The MPO activity confirmed the protective effects of KYP-2047 to reduce inflammation in CVI mouse model (**H**). Data represent the means of at least three independent experiments. One-way ANOVA followed by Bonferroni post-hoc. *** *p* < 0.001 versus Sham and ^###^ *p* < 0.001 versus CVI.

Histopathologic examination of lateral saphene vein subjected to ligation for 7 days revealed inflammatory cell infiltration, structural alteration of the tunica adventitia, and formation of focal epithelial edema, with the collapse of the whole vessel wall (Figure 5D, see histological score Figure 5G) compared to sham group (Figure 5A, see histological score Figure 5G). In control mice, treated with Simvastatin or with KYP-2047, for 7 days, no major modification of the vessel lumen was found, as well as no signs of tissue inflammation (Figure 5B,C, see histological score Figure 5G); treatment with KYP-2047, at the dose of 10 mg/kg, for 7 days significantly reduced hypertrophy of the tunica media, holding the collapse of the wall caused by the ligation (Figure 5F, see the histological score Figure 5G). The effect of KYP-2047 treatment was similar to the elective Simvastatin treatment (20 mg/kg), that prevented vessel lumen modification and tissue inflammation (Figure 5E, see the histological score Figure 5G). Moreover, the venous hypertension contributes to leucocytes accumulation and neutrophilic activation [43], that was measured in this study by analyzing the Myeloperoxidase (MPO) activity on veins samples. Particularly, a significant increase in MPO activity was observed in CVI-injured group compared to control (Figure 5H), while treatment with KYP-2047, like Simvastatin, significantly reduced the neutrophilic activation detected in veins subjected to ligation (Figure 5H).

### 3.6. Role of KYP-2047 Treatment in the Collagen Content Reduction and Elastin Replacement

**Figure 6:**
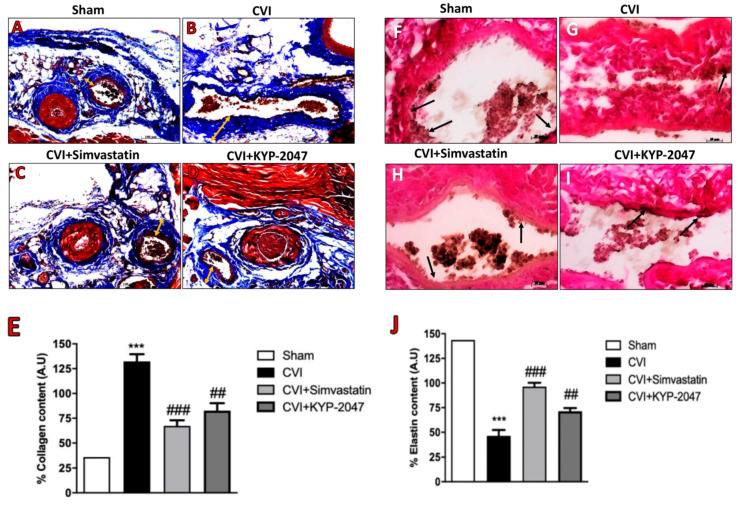

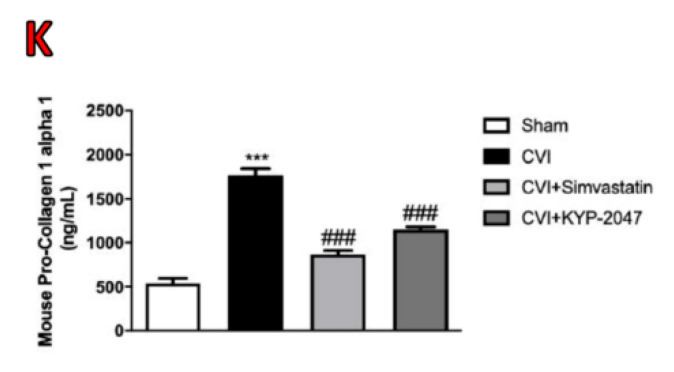
Treatment with KYP-2047 in the collagen content reduction and elastin increase. The degree of fibrosis, in CVI-provoked lesions, 7 days post-saphene vein ligation, was assessed by Masson trichrome staining and Van Gieson staining, revealing a fibrotic area stained blue larger than in control group (**B**), see collagen content score (**E**); (**A**), see collagen content score (**E**) and a reduced elastin content highlighted in red purple (**G**), see elastin content score (**J**); (**F**), see elastin content score (**J**). Treatment with KYP-2047 significantly reduced collagen depot, located in the tunica adventitia (**D**), see collagen content score (**E**) and increased the content of elastin fibers (**I**), see collagen content score (**J**) almost similarly to Simvastatin treatment (respectively (**C**), see collagen content score (**E**), (**H**), see elastin content score (**J**). Pro-collagen 1 levels were detected by ELISA kit, confirm the results observed by Masson trichrome staining (**K**). Orange arrows identified collagen content, while black arrows identified elastin content. Scale bar for figures A–D: 100 μm, magnification 10 ×. Scale bar for figures F–I: 20 μm, magnification 40 ×. Data represent the means of at least three independent experiments. One-way ANOVA followed by Bonferroni post-hoc. *** *p* < 0.001 versus Sham; ^##^ *p* < 0.01 and ^###^ *p*< 0.001 versus CVI.

The nature and distribution of venous disease surrounding the development of varicose veins correlate with collagen and elastin as important components of the vein walls that affect their function [44]. The vein wall resistance to stretch depends on the collagen fibers and it is known that in CVI pathology, varicose veins show increased collagenosis and dilated distal varicosities [44]. This study confirmed that in CVI-provoked lesions, 7 days post-saphene ligation, the degree of fibrosis, assessed by Masson trichrome staining demonstrated a fibrotic area stained blue that was larger in the CVI group (Figure 6B, see collagen content score Figure 6E) compared to control group (Figure 6A, see collagen content score Figure 6E). Treatment with KYP-2047 significantly reduced the blue staining, which represents collagen depot, located in the tunica adventitia (Figure 6D, see collagen content score Figure 6E), almost similar to Simvastatin treatment (Figure 6C, see collagen content score Figure 6E). The collagen content in veins samples was also investigated by kit ELISA for pro-collagen I, highlighting a significant increase of collagen depot in CVI-injured groups compared to control mice (Figure 6K), also confirming the role of KYP-2047 and Simvastatin to reduce this collagen accumulation (Figure 6K). Contrarily, when the vessel wall is stretched, elastin generates a retractive force that opposes the lengthening force caused by pressure in the lumen vessel [45]. In this study, we observed a decreased amount of elastin in veins from SVL-damaged group, compared to control group (respectively, Figure 6F,G, see elastin content score Figure 6J); instead, treatment with KYP-2047, albeit to a lesser extent than Simvastatin treatment, notably increased elastin content thus preventing the collapse of lumen vessel (respectively, Figure 6H,I, see elastin content score Figure 6J).

### 3.7. Role of KYP-2047 Treatment in Preventing Mast Cell Degranulation

**Figure 7:**
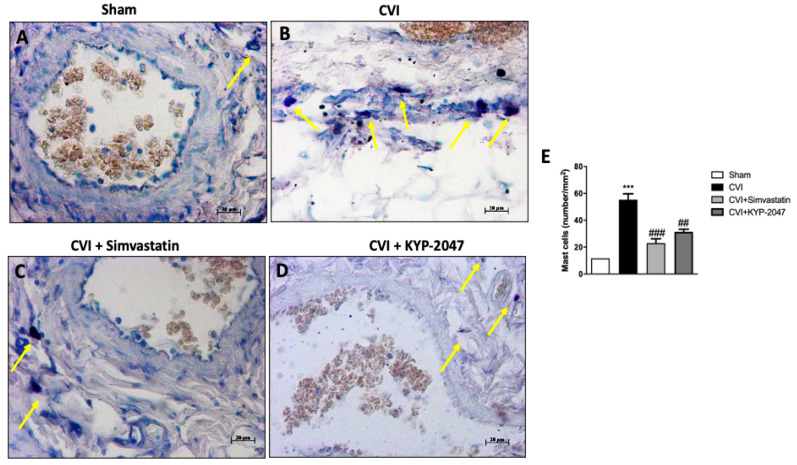
Treatment with KYP-2047 treatment on mast cells. Mast cells were evaluated in saphene veins tissues by staining with toluidine blue. Compared with sham animals, the mast cells number in CVI-damaged group was significantly increased (respectively, (**A**), (**B**), see graph (**E**). Neutrophilic accumulation was appreciably reduced by KYP-2047 or Simvastatin treatment (respectively, (**C**,**D**), see graph Figure 3E). Yellow arrows indicated the mast cells presented in the area. Scale bar for figures F-I: 20 μm, magnification 40 ×.Data represent the means of at least three independent experiments. One way ANOVA followed by Bonferroni post-hoc. *** *p* < 0.001 versus Sham; ^##^ *p* < 0.01 and ^###^ *p* < 0.001 versus CVI.

Increased infiltration of activated mast cells has been recently implicated in the pathophysiology of varicose veins [46]; mast cells produce and release various kinds of vasoactive substance, including histamine, tryptase, prostaglandins, leukotrienes, and cytokines, that enhance local vasopermeability, leading to intimal thickening [47]. Mast cells were best identified in saphene veins tissues by their characteristic numerous metachromatic granules by using toluidine blue stain (Figure 7). Compared with the control group, the number of mast cells in CVI-damaged group was significantly increased (respectively, Figure 7A,B, see graph Figure 7E). Mast cells degranulation was appreciably reduced with KYP-2047 or Simvastatin treatment (respectively, Figure 7D,C, see graph Figure 7E).

### 3.8. Evaluation of KYP-2047 in a Saphene Vein Block Culture Study

**Figure 8:**
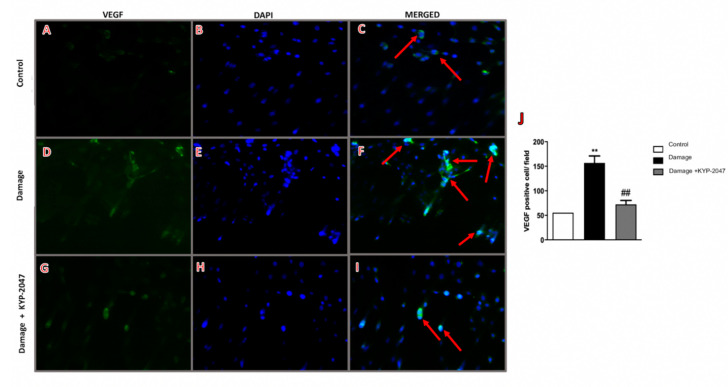
VEGF expression on saphene vein from tissue block culture study. Immunofluorescence analysis, on tissue block culture study, revealed an increase in VEGF expression in VSMCs from CVI-tissue block (**D**–**F**), see VEGF positive score (**J**) compared to control cells (**A**–**C**), see VEGF positive score (**J**). POP inhibition as treatment on CVI-damaged veins positively modulated angiogenesis as seen by VEGF expression in CVI + KYP-2047 tissue block group (**G**–**I**), see VEGF positive score (**J**). Red arrows refers to VEGF-positive cells merged with DAPI. Scale bar for figures F–I: 20 μm, magnification 40× Data represent the means of at least three independent experiments. One-way ANOVA followed by Bonferroni post-hoc. ** *p* < 0.01 versus Control; ^##^ *p* < 0.01 versus CVI.

**Figure 9:**
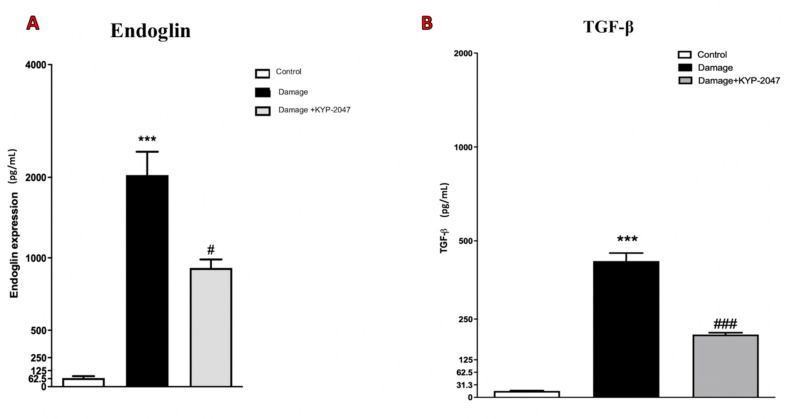
KYP-2047 treatment on TGF-β1 and Endoglin levels on saphene vein from tissue block culture study. The content of endoglin and TGF-β1 were evaluated by ELISA kits, revealing an increase in damaged group compared to control (**A**,**B**), while KYP-2047 treatment notably reduced this increase (**A**,**B**). Data represent the means of at least three independent experiments. One way ANOVA followed by Bonferroni post-hoc. *** *p* < 0.001 versus Sham; ^#^ *p* < 0.05 and ^###^ *p* < 0.001 versus CVI.

To underline the modulatory action of the KYP-2047 on angiogenesis, the in vivo study was repeated performing a new study from tissue block culture. The VSMCs obtained from tissue block culture study have been tested by immunofluorescence analysis for VEGF, considering that an up-regulation of this marker in the skin of patients with CVD has been demonstrated [48]. In this study, VEGF expression significantly increased in VSMCs from CVI-tissue block (Figure 8D–F, see VEGF positive score Figure 8J compared to control cells (Figure 8A–C, see VEGF positive score Figure 8J). Treatment with KYP-2047 on CVI-damaged veins reduced angiogenesis to bring it back to physiological conditions (Figure 8G–I, see VEGF positive score Figure 8J). Moreover, endoglin, plays an important role in vascular development, regulating angiogenesis through the interaction with VEGF receptor [49]. In this study, endoglin content was evaluated through an ELISA kit performed on supernatants of VSMCs cell extracted from tissue blocks; a notable increase in endoglin expression (pg/mL), was observed in CVI samples, compared to control group (Figure 9A), while VSMCs treated with KYP-2047 showed a reduced endoglin content, released in supernatants (Figure 9A). Furthermore, as Endoglin the co-receptor of the TGF-β, this marker was evaluated by ELISA kit also on VSMCs cell extracted from tissue blocks, observing for the first time a significant increase in TGF-β quantity (pg/mL) in CVI samples compared to control (Figure 9B), while treatment with KYP-2047 decreased this content (Figure 9B).

## 4. Discussion

Chronic venous disease (CVD) is a very common problem [50], with an higher prevalence in Western countries where it already consumes up to 2% of the healthcare budgets [51]. However, because the most common manifestation of the pathology is represented by varicose veins, which begin as a result of incompetent valves and augmented venous pressure, the term CVI often symbolizes the full spectrum of manifestations of CVD, from simple telangiectases to skin fibrosis and venous ulceration [52]. Generally, when pressure is increased and return of blood is impaired, the onset of venous pathology is insured, resulting from valvular incompetence of axial deep or superficial veins, venous tributaries, venous obstruction, or a combination of these mechanisms [53]. The manifestations of CVI may be viewed in terms of a well-established clinical classification scheme; particularly, “The Clinical, Etiology, Anatomic, Pathophysiology” (CEAP) classification was developed by an international consensus conference to provide a basis of uniformity in reporting, diagnosing, and treating CVI [30]. The management of CVI starts with conservative measures to reduce the symptoms and prevent the development of secondary complications and progression of disease, then move on to further treatment if conservative measures fail or provide an unsatisfactory response [7]. Despite this, suitable pharmaceutical therapies to totally stem the pathology have not been explored to date and given the prevalence and socioeconomic impact of CVD, an understanding of new therapeutic options is warranted.

Recent findings indicate that inflammation and angiogenesis alterations greatly concur to the onset of CVI [31]; particularly, inflammatory tissue is often hypoxic and hypoxia can induce angiogenesis through upregulation of factors such as vascular endothelial growth factor (VEGF) and chemokines can both promote angiogenesis and stimulate the recruitment of inflammatory cells [54]. Recent findings indicate that peripheral POP may be involved in the inflammation [55] and in angiogenesis [56]. KYP-2047 is a very potent selective inhibitor of POP [57]. Thus, based on these evidences, the aim of this study was to evaluate the protective effect of KYP-2047 to counteract inflammatory and angiogenetic process involved in the pathophysiology of CVI through an in vivo mouse model of CVI.

First, the expression of POP was evaluated in this CVI mouse model, highlighting an increase expression of POP enzyme in vascular pathology, also confirming the efficiency of KYP-2047 to inhibit POP activity and, for the first time, to negatively modulate the expression of POP.

A critical inflammatory and angiogenetic mediator is represented by TGFβ-1, an important factor involved in regulating leucocyte and fibroblast recruitment and ECM remodeling, by both stimulating fibrogenesis and deposition of collagen [58]. TGF-β1 is able to act as a promoting and an inhibitory factor of angiogenesis and it is known to maintain a balance between apoptosis and cellular dysfunction, having a pivotal role in vessel remodeling during pathogenesis of vascular disorders [59]. In this study, an increased expression of TGFβ-1 was observed in damage conditions, while the treatment with KYP-2047 could greatly reduce the cascading events associated with the expression of TGFβ-1. TGF-β1 and IL-8 are important regulators of inflammation-induced angiogenesis [60], so the angiogenesis mechanisms involved in CVI disorders were also investigated evaluating IL-8, known as a potent factor, intricated in normal physiological processes and abnormal mechanisms, directly enhancing the endothelial cell survival and regulating vascular tone [61]; in this study, we confirmed an increased expression of IL-8 in vein samples compared to control, while treatment with Simvastatin and KYP-2047 reduced this increment.

Furthermore, the role of angiogenesis is correlated to the amount of α-SMA of primary varicose veins and various studies highlighted the relation between α-SMA and VEGF in vascular wall injury [59]. In this work, a significant reduction in VEGF/α-SMA ratio was observed in KYP-2047 and Simvastatin-treated groups, compared to CVI-damaged mice. VEGF primarily exerts its effect through the production of vasodilatory mediators, particularly causing an overproduction of NO [41]; NO release is associated with varicosity development [62] as well as a critical role for eNOS in controlling the magnitude of the acute inflammatory response for regulating microcirculatory endothelial barrier function [63]. In this study, we confirmed an increased augment of eNOS production in CVI-damaged group, closely associated with a significative decrement in Simvastatin and KYP-2047 groups.

The production/activity of vasodilatory mediators such as nitric oxide depends by cytokine-induced activation in vascular pathologies [64]. The state of inflammation in patients with venous disease highlighted that leucocytes sequestering leads to the activation of blood white cells, resulting in the release of free radicals, histamine, and neutrophil chemoattractants; the actions of these substances destroy the endothelial layer of the vessel and their basement membranes, increasing the vascular permeability and disturbing microcirculatory flow. Furthermore, the movement of white blood cells from the adventitia to the medial layer and the upper part of the vein wall, helped trigger the release of proinflammatory cytokines [65]. We demonstrated the capacity of KYP-2047, likewise Simvastatin treatment, to counteract the expression of pro-inflammatory markers, IL-1β and TNF-α, in SVL-damaged animals, highlighting the role of POP inhibitor to reduce the inflammation process in CVI.

The inflammatory response, the attraction of neutrophils, and the damage to veins are factors that perpetuate venous insufficiency and contribute to modify veins structure [66]. In this study, we observed by in vivo model of CVI that treatment with KYP-2047 significantly reduced the structural and morphological alterations provoked by ligation of saphene veins, decreasing the intimal and adventitia fibromuscular plaques, counteracting neutrophilic accumulation and preventing the whole wall vein collapse. Moreover, recent studies suggest that statins improve the microvascular function, effectively by inhibiting the development of varicose veins, thus representing a treatment option to prevent the growth of varicose veins and to limit the chances of recurrence after varicose vein surgery [21]. In this study, treatment with KYP-2047 showed to restore the histological alterations caused by vein ligation, similarly to the benefits observed by treatment with Simvastatin. Furthermore, as leucocytes become “trapped” in the circulation of the leg during periods of venous hypertension produced by sitting or standing, studies of the plasma levels of neutrophil granule enzymes show that these are increased during periods of venous hypertension, suggesting that this causes activation of the neutrophils. We confirmed neutrophilic activation in this CVI mouse model evaluating the MPO activity, that was significantly reduced by KYP-2047 or Simvastatin treatments.

Moreover, alterations on the connective tissue concentration and smooth muscle are visible in vascular disease [67]. In fact, the venous systems structure follows a pattern in concentric layers: intima, media, and adventitia, which undergo a modification of structural components of the vessel wall in conditions of altered venous flow, associated to loss of tone and the subsequent venous dilatation [68]. The Masson’s trichrome stain revealed in red the smooth muscle fiber to smooth muscle fiber (SMF), while dyed bluish corresponded to the extracellular matrix (ECM), in which many elements such as collagen and elastin are arranged [69]. Some studies connected reflux with weakening of the venous walls, which may be due to an imbalance in the content of collagen and elastin in the vein [62]. In this study, the distribution and the relationships between SMF and ECM were impaired in CVI-damaged mice, thus reducing the tone and the progressive dilatation of the vein wall, compared to the control mice; while, treatment with KYP-2047 significantly reduced the disparity between the collagen and elastin content, similarly to Simvastatin treatment. The KYP-2047 modulation on collagen fibers in CVI was also confirmed by quantifying Pro-collagen 1, as the major vascular fibrillar collagen [63].

Recent findings suggest that inflammatory processes is crucial for the development of inept valves and vein wall remodeling; particularly, it is postulated that varicose lesions showed a greater extent of mast cell infiltration whereas baseline control veins had a smaller number of mast cells [70]. This research underlined the efficacy of KYP-2047 treatment to counteract mast cell degranulation phenomenon, compared to only CVI-damaged group, as noted by toluidine blue staining.

During the resolution phase of inflammation, the endothelial to mesenchymal (EndoMT)-mediated remodeling commonly occurs, bringing the endothelial cells from different vascular beds to respond differently to inflammatory stimuli [71]. Most of the signaling networks that are commonly utilized during epithelial-mesenchymal transition (EMT) are also responsible for EndoMT, seeing the active participation of TGF-β1 [72]. In this study, we confirmed for the first time, in a tissue block culture study, a significant increase in TGF-β1 quantity in VSMCs from CVI-tissue block, which might suggest a TGF-β-related induction of endothelial-to-mesenchymal transition. Furthermore, an important role in vascular development and tumor-associated angiogenesis is represent by Endoglin [73], a transmembrane auxillary receptor for TGF-β that is predominantly expressed on proliferating endothelial cells. It is known that endoglin promoted VEGF-induced tip cell formation, mechanistically, interacting with VEGF receptor (VEGFR) [49]. The demonstrated changes in mRNA expression, as well as in the contents of VEGF receptors, in the wall of varicose veins is accepted as one of the reasons for the clinical symptoms of the disease and can predispose to its progression [70]. In this research, we confirmed that the over-expression of both VEGF and endoglin in VSMCs cells from saphene veins subjected to binding, underlying as treatment with KYP-2047, inhibiting POP, significantly decreased these increases in angiogenesis markers.

CVI is a disease characterized by numerous risk factors, some of which suggest that disease can occur because of congenital valve or vessel abnormalities, but it most commonly occurs when the valves of the deep veins are damaged as a result of deep venous thrombosis, as well as, the expression of specific cardiovascular markers could contribute to the onset of the pathology. So, a limitation for this in vivo *study,* is represented by the difficulty in paying attention to all diagnostic markers that could contribute to the beginning of CVI.

Despite this, the results obtained in in vivo studies and confirmed in tissue block culture study, suggest a pivotal role of POP inhibition in the hypertrophy of the venous wall, although the exact mechanism leading to venous wall dilatations remains to be elucidated. Furthermore, the action of the KYP-2047, although it is similar to that of Simvastatin, today considered as the treatment most often used in CVD, has the extra gear because it is able to act in a targeted and effective manner.

## 5. Conclusions

Based on these results, POP inhibition due to KYP-2047, could represent a remarkable strategy to counteract the negative effects associated with vascular alterations. These data suggest a strong anti-inflammatory potential of KYP-2047 associated to its modulatory role on angiogenesis, that contribute to positively modulate CVI, offering new therapeutic target tools in the management of vascular pathologies.

## Data Availability

The datasets used and/or analyzed during the current study are available from the corresponding author on reasonable request.
